# Systematic review: *Xenopus laevis* as a model for ophthalmic development and disease research

**DOI:** 10.3389/fmed.2025.1545958

**Published:** 2025-10-24

**Authors:** Qiaoyu Li, Yun Feng, Xuechen Zhu

**Affiliations:** ^1^Department of Ophthalmology, Peking University First Hospital, Beijing, China; ^2^Department of Pediatrics, Peking University First Hospital, Beijing, China; ^3^Department of Human Anatomy, Histology and Embryology, School of Basic Medical Sciences, Peking University Health Science Center, Beijing, China; ^4^Neuroscience Research Institute, Peking University, Beijing, China

**Keywords:** *Xenopus laevis*, eye, development model, disease model, ophthalmology

## Abstract

Model organisms have played a pivotal role in ophthalmic research, providing essential platforms to investigate eye development, regeneration mechanisms, and disease pathology. Recent advancements in gene editing technologies and experimental methodologies have enabled the successful simulation of various human eye diseases, including glaucoma, retinal degeneration, and corneal disorders in model systems. These models have significantly advanced the understanding of the molecular and cellular mechanisms underlying ocular diseases and facilitated the screening and validation of potential therapeutic agents. *Xenopus laevis* (*X. laevis*) has emerged as an ideal system for developmental biology research due to its rapid embryonic development, transparent embryos, and ease of observation and manipulation. Its fully sequenced genome allows precise genetic modifications, including gene knockout, knock-in, and expression regulation studies. In ophthalmic research, *X. laevis* is widely used for studying eye development, disease modeling, and ocular structure. Its accessible embryonic stages and well-characterized eye development make it a valuable model for retinal disease investigations. This review systematically summarizes the applications, construction methods, and research significance of *X. laevis* models in eye development, disease modeling, and drug screening. It provides an in-depth perspective on the utility of *X. laevis* in foundational ophthalmic research, offering insights to guide future studies.

## 1 Introduction

In ophthalmic basic research, model organisms have provided a crucial experimental platform for exploring eye development, regenerative mechanisms, and disease pathologies. In recent years, researchers have leveraged gene-editing technologies and innovative experimental methods to successfully replicate various human ocular diseases, including glaucoma, retinal degeneration, and corneal pathologies, in model animals. These models not only facilitate a deeper understanding of the molecular and cellular mechanisms underlying ophthalmic diseases but also serve as essential tools for screening and validating potential therapeutic drugs.

With its fully sequenced genome, Xenopus enables studies involving gene knockout, gene knock-in, and gene expression regulation. Due to its rapid embryonic development and transparent embryos, *X. laevis* provides a simple, cost-effective, and highly observable system readily amenable to manipulation. In ophthalmic research, particularly in studies of eye development, ocular disease models, and ocular structure, the transparency of its embryos and the ease of manipulating eye development make it an optimal model for rapidly investigating disease mechanisms. *X. laevis* is therefore not merely a technical convenience but a powerful complementary model for dissecting fundamental biological mechanisms in a high-throughput and accessible manner.

This review provides a systematic overview of the applications, construction methodologies, and research significance of *X. laevis* models in studies of eye development, disease modeling, and drug screening. It offers a comprehensive retrospective and in-depth insights into the use of Xenopus in ophthalmic research, aiming to inspire future directions in the field.

## 2 Model organisms in ophthalmic disease research

**Figure 1:**
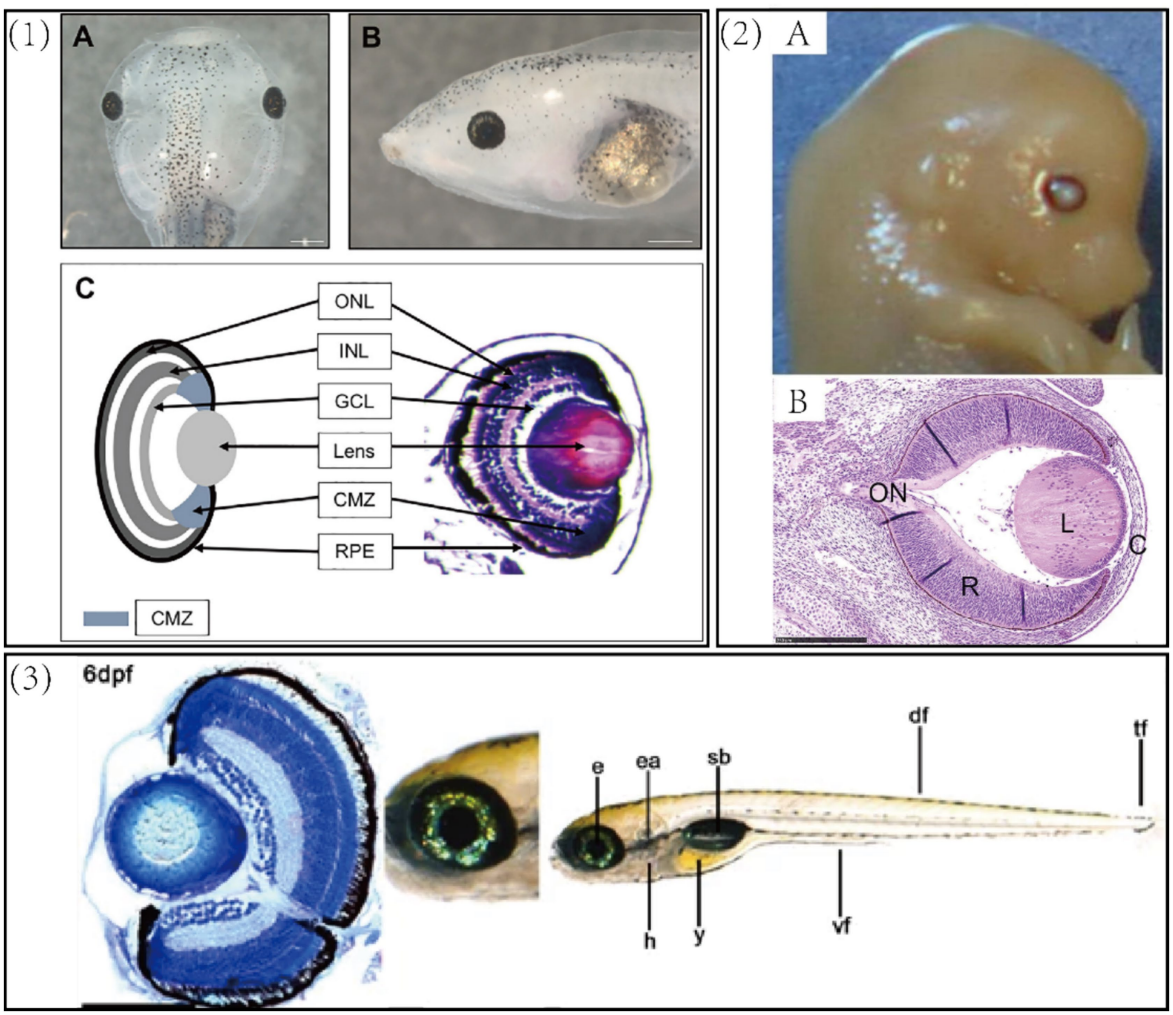
(1) *Xenopus laevis* tadpole. **(A)** A dorsal view of a 6-day old swimming tadpole. **(B)** A side view of the same tadpole. **(C)** A schematic (left) and transverse section (right) of the tadpole retina. ONL, outer nuclear layer—rod and cone photoreceptors; INL, inner nuclear layer—bipolar, horizontal, and amacrine cells; GCL, ganglion cell layer—retinal ganglion cells and amacrine cells; CMZ, ciliary margin zone—containing retinal stem cells, blue regions at the periphery of the lens. The outer layer covering the eye is the outer corneal epithelium and the layer directly adjacent to the lens is the inner corneal layer. Scale bar = 500 μm (111). (2) **(A)** Gross morphology of mouse eyeball. **(B)** Cross-sectional view of the eye (H&E staining). Bar = 250 um. C, cornea; L, lens; ON, optic nerve; R, retina (112). (3) Gross morphology and cross-sectional staining of zebrafish eye. e, eye; ea., ear; h, heart; y, yolk sac; sb, swimbladder, df, dorsal fin; tf, tail fin. Scale bars = 100 μm (113).

Of the models used to study visual field specification and human ocular pathologies (Figure 1), the mouse remains the premier mammalian model due to its 99% gene conservation rate with humans and well-annotated mutant lines that facilitate the study of systemic gene impacts (1–3). However, its significant limitation for studying fundamental developmental concepts is the extreme difficulty or impossibility of administering interventions during early fetal stages. In contrast, *Xenopus laevis* has emerged as an ideal complementary model and powerful tool for rapid, cost-effective mechanistic discovery, offering a unique combination of advantages not fully replicated in other systems.

While other models such as zebrafish and chicken are also employed, they lack the specific combination of features that make *X. laevis* particularly special for mechanistic research. Zebrafish share high genetic conservation and transparent embryos but possess duplicated genes that can complicate phenotypic analysis (4). As an amphibian, *X. laevis* exhibits closer parallels to mammals than to zebrafish in key physiological processes, such as regulating osmotic pressure and undergoing metamorphosis (5). While zebrafish share high genetic conservation and transparent embryos ideal for live imaging, but their relatively small size limits the scale and ease of microsurgical manipulations that are possible in the much larger and more robust *X. laevis* embryos. The chicken embryo allows for direct observation but is limited by its evolutionary distance and genetic tools (6, 7). Drosophila is powerful for genetic screens but has limited utility for human ocular diseases due to vast physiological differences (8).

The core special of *X. laevis* lies in its ability to seamlessly integrate powerful genetic manipulation with embryonic accessibility at any developmental stage—a capability rare in other vertebrate models. As a tetrapod, it shares 79% genetic similarity with humans and comparable principles of eye development (9–12). Crucially, its embryos can be easily manipulated and induced at any stage, which is essential for validating developmental concepts in a living system. Specifically: (1) Their large size and transparency facilitate microinjection and single-cell transplantation to study tissue interactions, offering a strong self-control model at the 2- or 4-cell stage (10). (2) The Founder generation 0 (F0) reverse genetics approach enables phenotypic analysis in transgenic larvae without time-consuming breeding, allowing for rapid gain- and loss-of-function studies and the swift generation of transgenic models (9, 13). (3) Researchers can further integrate multi-omics approaches with XenBase resources to decipher developmental transcriptional networks.

It is important to note that gene duplicates in Xenopus may require careful interpretation of phenotypic severity due to potential compensatory mechanisms, and Morpholino use warrants controls (e.g., p53 co-injection) to minimize off-target effects (14). Nonetheless, for research aimed at rapidly and cost-effectively decrypting mechanisms, screening pathogenic genetic variants, and validating developmental hypotheses, the unique combination *X. laevis* offers—high physiological relevance, exceptional experimental accessibility, and speed—establishes it as a particularly powerful and efficient model system (Table 1).

**Table 1:** Overview of diseases and genes.

Disease name	Key gene(s)	Inheritance pattern	Animal model	Gene manipulation method	Phenotypic results/human pathology correlation
Aniridia	*PAX6*	Autosomal Dominant	Xenopus (TALEN knockout), Mouse (conditional KO)	TALENs, CRISPR-Cas9	Iris developmental absence, corneal abnormalities; consistent with human *PAX6* mutation phenotypes
Retinitis Pigmentosa (RP)	*RHO* (P23H/T17M, etc.)	Autosomal Dominant/Recessive	Xenopus (transgenic), Mouse (KO)	CRISPR, transgenic overexpression	Rod cell degeneration, photoreceptor outer segment abnormalities; mimics progressive vision loss in human RP
Stargardt Macular Dystrophy	*ELOVL4*	Autosomal Dominant	Xenopus (mutant overexpression)	Morpholino, overexpression	Mislocalization of photoreceptor outer segments to Golgi, retinal degeneration
Congenital Cataract	*GJA8* (Cx50), *MIP*	Autosomal Dominant	Xenopus (CRISPR, morpholino)	CRISPR-Cas9, morpholino knockdown	Lens opacity, defective fiber cell differentiation; matches human congenital cataract phenotypes
Congenital Stationary Night Blindness (adCSNB)	*PDE6B*	Autosomal Dominant	Xenopus (heterologous expression)	mRNA microinjection	Persistent activation of rod cell signaling, impaired adaptation
Retinoblastoma	*RB1*	Autosomal Recessive	Xenopus (CRISPR chimera)	CRISPR-Cas9	Retinal tumor formation, mimics biallelic *RB1* inactivation in children
Corneal Endothelial Dystrophy	*SLC4A11*	Autosomal Recessive	Xenopus oocytes (heterologous expression)	mRNA microinjection	Endothelial water transport defects causing stromal edema; correlates with CHED and FECD phenotypes
Cornea Plana	*RAD21* (C1348T)	Autosomal Dominant	Xenopus embryos (mRNA injection)	mRNA microinjection	Disorganized corneal stromal collagen fibers, mimics human cornea plana
Galloway-Mowat Syndrome	*TP53RK*	Autosomal Recessive	Xenopus (morpholino knockdown)	Morpholino oligonucleotide	Ocular and cranial developmental abnormalities, rescuable by human wild-type *TP53RK*
Joubert Syndrome (Ciliopathy)	*PIBF1*	Autosomal Recessive	Xenopus (CRISPR)	CRISPR-Cas9	Neural tube cilia formation defects, retinal developmental abnormalities
NCMD Macular Dystrophy	*PRDM13/IRX1* regulatory region	Autosomal Dominant	Xenopus (enhancer activity analysis)	Luciferase reporter gene	Non-coding mutations affecting retinal developmental regulation
Stargardt-like Dystrophy	*PROM1*	Autosomal Dominant/Recessive	Xenopus, Mouse	CRISPR	Photoreceptor outer segment morphological abnormalities, resembles cytochalasin D-treated phenotypes
Cone-Rod Dystrophy	*PrCAD*	Autosomal Recessive	Xenopus, Zebrafish	Morpholino knockdown	Outer segment basal structure defects, consistent with human cadherin mutation phenotypes
X-linked Megalocornea (XMC)	*CHRDL1*	X-linked Dominant	Xenopus (CRISPR)	CRISPR-Cas9	Anterior segment enlargement, corneal dystrophy, BMP signaling pathway abnormalities
Nuclear Cataract	*DNASE2B*	Autosomal Recessive	Xenopus (CRISPR)	CRISPR-Cas9	Defective lens fiber cell nuclear degradation, improvable by 4-phenylbutyrate

## 3 Current research directions

### 3.1 Ocular development and eye defects

Previous research has highlighted that embryonic intervention studies are essential for defining the timing of ocular induction, elucidating early regulatory factors in eye development, and clarifying the molecular pathways underlying ocular malformations (10, 15, 16). In *X. laevis*, ectopic *several eye-field transcription factors (EFTFs)* require *Otx2* to induce the eye field, revealing *Otx2*’s permissive role in early eye specification. By transplanting fluorescently labeled tissue expressing *EFTFs* and *Otx2* into various regions of host embryos, researchers observed organized, functional eye-like structures, indicating that these factors alone can stimulate and coordinate eye morphogenesis (16–18). In the early stages of embryogenesis (neural plate stage, approximately 12 h post-fertilization), the *Pax6* gene is first expressed, serving as the “master regulator” that initiates eye field formation (19). Shortly thereafter, *Rx1* expression begins, co-regulating the early development of the ocular region alongside *Pax6* (16). *Six3* also plays a crucial role at this stage, cooperating with *Pax6* and *Rx1* to further define and refine the formation and differentiation of the eye field (20). As development progresses to the optic vesicle formation stage (around 24 h post-fertilization), fibroblast growth factor (FGF) signaling becomes active, promoting the extension of the optic vesicle and the formation of the optic cup (21). Concurrently, bone morphogenetic protein (BMP) signaling operates through distinct pathways to ensure the optic cup’s correct shape and positioning (22). Additionally, Sonic Hedgehog (Shh) signaling maintains symmetry and proper morphology in the eye field by modulating cell proliferation and differentiation (23).

Mutations in *Pax6* lead to ocular malformations in both vertebrates and invertebrates, with *PAX6* mutations in humans causing aniridia (24). In Xenopus, embryos injected with transcription activator-like effector nucleases (TALENs) targeting *pax6a* and *pax6b* in over 85% of cells displayed severe disruptions in eye formation and various ocular malformations, including microphthalmia and tissue deficiencies (25). In partial loss-of-function *Pax6* mutants, early eye development appears relatively normal, but by the tadpole stage, the iris shows developmental deficiencies similar to the typical phenotype of aniridia in human *PAX6* mutation carriers (26). These mutant tadpoles also exhibit other ocular anomalies, such as cataracts and corneal defects, which are frequently observed in human aniridia patients (27).

### 3.2 Retinal development and disease model

#### 3.2.1 Retinal development model

The neural retina is composed of six distinct cell types—ganglion cells, amacrine cells, horizontal cells, photoreceptors, bipolar cells, and Müller glial cells—that are arranged in a stereotypical layered structure conserved across all vertebrates (12). *X. laevis* is a well-established model for studying neurodevelopment, particularly retinal cell fate determination (28–31). During Xenopus eye development, eye field specification is a critical step that governs the conversion of neuroectodermal cells into retinal cells. Initially, at the blastula stage, most animal pole cells have the potential to form retinal cells. By stage 12.5, when gastrulation is almost complete and the neural plate is forming, cells in the anterior region of the neural plate (the eye field) will differentiate into retinal tissue and eyes if cultured in a neutral environment or transplanted to the ventral side of an embryo. Hours before stage 12.5, cells transplanted from the same area do not form retina or eyes. This series of molecular changes, occurring within hours, enables neuroectodermal cells in frogs to specialize into retinal cells, a process termed eye field specification. Studying this stage can enhance understanding of the molecular mechanisms behind eye field specialization, thus providing an important model system for retinal development and regeneration research (14). Following optic cup formation (~48 h), the *Notch* pathway coordinates progenitor proliferation and differentiation (e.g., via *Atoh7* and *Ngn2*) to generate retinal cell diversity (32, 33). Beyond the major molecular players in eye field specification, key regulators like *KDM7A* and *Rax/Rx1* dynamically control this process (34). Regulated by *Rax/Rx1*, *KDM7A* is expressed early in Xenopus development and is temporally and spatially regulated in the central nervous system and eye (35). *KDM7A* expression is dynamic during embryonic development; overexpression disrupts late retinal development, hindering ganglion cell development and promoting horizontal cell formation, potentially contributing to the molecular mechanism regulating the spatiotemporal generation of retinal neuron subtypes (36).

##### 3.2.1.1 Key proteins in retinal specialization

Opsins can detect light and perform imaging or non-imaging tasks, yet little is known about the retinal expression cells, developmental onset, and photoactivation of neural opsin proteins (37). Neural opsins (opn5, 6a, 6b, 8) exhibit stage-specific expression: opn5 and opn8 initiate at stage 37/38, coinciding with retinal circuit activation. Once the retinal circuitry connects to the brain, opn5 mRNA localizes in multiple retinal cell types, including bipolar cells (~70–75%), amacrine cells (~10%), and retinal ganglion cells (~20%), while opn8 is present in amacrine cells (~70%) and ganglion cells (~30%). Opn6a and opn6b mRNA emerge in newly formed photoreceptors by stage 35 and co-localize in rods and cones at stage 37/38. In the mature larval retina (stage 43/44), opn6a and opn6b mRNA preferentially localize in rods and cones, respectively, with newly formed photoreceptors bordering the proliferative ciliary marginal zone co-expressing both genes. The majority of retinal ganglion cells expressing neural protease show c-fos expression under light stimulation, and more than half of intermediate neurons expressing neural protease do so as well (38).

Post-translational modification is critical for the proteins involved in eye field specification, with presenilin (PS)/*γ*-secretase acting as the catalytic component of γ-secretase to cleave single-pass transmembrane proteins essential for development, such as *Notch*, netrin receptor DCC, cadherins, drebrin-A, and EphB2 receptors. Studies have shown that inhibiting PS expression in post-synaptic tectal neurons in the Xenopus tadpole retinotectal circuit diminishes visual avoidance behavior and weakens synaptic transmission, significantly reducing NMDA receptor (NMDAR) and AMPA receptor (AMPAR)-mediated currents. Further research indicates that expressing the C-terminal fragment of the EphB2 receptor rescues the NMDAR-mediated response reduction; this fragment, typically cleaved by PS/*γ*-secretase, is known to upregulate synaptic NMDAR, suggesting that normal PS function is essential for the proper formation and potentiation of retinotectal synapses via EphB2 cleavage (39).

##### 3.2.1.2 Key protein mutations affecting retinal and neurodevelopment

Recent studies have shown that the absence of certain key proteins can lead to severe retinal neurodevelopmental abnormalities, even if their role in eye field specification remains unclear. Nitric Oxide Synthase Interacting Protein (Nosip), associated with various human diseases, is essential for the development of neural precursor tissues, such as the eye and neural crest cells. Research indicates that Nosip gene expression occurs in the developing eye system and neural crest cells of *X. laevis*, and Nosip inhibition results in severe eye formation defects in both mice and Xenopus. In Nosip-deficient Xenopus embryos, retinal layering and dorsal-ventral patterning of the retina are disrupted. Marker gene analyses (e.g., *rax*, *pax6*, and *otx2*) reveal that induction and differentiation of the eye field are hindered. Nosip deficiency also impairs cranial cartilage structure, inhibits neural crest cell induction and migration, and affects downstream factors of retinoic acid signaling, such as *foxc1* (40). The Fezzin family member Nedd4 Binding Protein 3 (*N4BP3*) is expressed in the neuroectoderm of Xenopus embryos, including the eye, brain, and neural crest cells. Knockdown of *N4BP3* in anterior neuroectoderm leads to severe developmental defects in eye, brain, and neural crest-derived cranial cartilage structures. *N4BP3* deficiency significantly reduces the expression of eye and brain-specific marker genes, decreases neural crest cell migration, and impacts cell apoptosis and proliferation, suggesting that *N4BP3* is essential for early anterior neural development in vertebrates, consistent with findings linking human *N4BP3* gene disruption to neurodevelopmental disorders (41).

#### 3.2.2 Retinal disease models

##### 3.2.2.1 Retinitis pigmentosa

Retinitis pigmentosa (RP) is a common hereditary retinal dystrophy characterized by the gradual degeneration of rod cells, followed by the non-cell-autonomous death of cone cells, progressive vision loss, and eventual blindness (42). Over 200 mutations in 50 different genes are associated with this disease, but there is currently no cure (43, 44). Rhodopsin, a cilia-specific G-protein-coupled receptor (GPCR), is transported with high fidelity to the outer segment (OS) of vertebrate rod cells; disruption in rhodopsin trafficking results in photoreceptor apoptosis and RP-associated blindness (45, 46). Mutations in the rhodopsin gene account for approximately 10% of RP cases worldwide. These mutations can cause endoplasmic reticulum (ER) retention and cell death, mislocalization, constitutive activity, or impaired protein–protein interactions. Using a rhodopsin-based gene-editing model in Xenopus, researchers discovered that loss of rhodopsin function leads to significant rod cell degeneration, displaying ultrastructural defects in the outer and inner segments, including vesiculation in the OS and rapid phagocytosis by retinal pigment epithelium (RPE), with occasional cell death, followed by morphological deterioration of cone cells (47, 48). Expression of the autosomal dominant rhodopsin mutation Ter349Glu in Xenopus revealed that this mutation, which adds 51 amino acids to the C-terminus of rhodopsin, causes mislocalization, OS developmental abnormalities, and disk formation defects (49). Studies in Xenopus expressing truncated rhodopsin mutants identified the CCGKN motif (amino acids 322–326) as critical for localization fidelity; additional signals in this region promote mislocalization. While the VXPX motif offsets mislocalization signals, enhancing ciliary targeting and transport (50).

The P23H mutation in rhodopsin is a major cause of autosomal dominant RP. Expression of bovine P23H rhodopsin in Xenopus showed that light-induced degeneration of P23H rods occurs in at least two stages: first, impaired phototransduction, followed by morphological changes (51). The P23H mutation triggers light-dependent degeneration through autophagy: cells degrade misfolded rhodopsin or secretory pathway debris, ultimately leading to apoptosis (48, 52). Visualizing autophagic structures in transgenic Xenopus rod cells expressing the dual-fluorescent autophagy marker mRFP-eGFP-LC3 revealed autophagosome maturation and degradation over 28 h, with more autophagosomes in rods expressing misfolded RHO P23H under light-induced autophagy enhancement (53). Expression of glycosylation-deficient rhodopsin mutants (T4K and T17M) in Xenopus explored light-exacerbated retinal degeneration mechanisms, finding that additional disulfide bonds increased thermal stability and reduced retinal degeneration. These mutants exhibited reduced toxicity when lacking chromophore binding sites or when vitamin A was absent in the diet; however, active conformation of these mutants, even in darkness, led to retinal degeneration, suggesting that vitamin A supplementation may be ineffective or harmful in glycosylation-deficient RP genotypes (54). Studying the effects of valproic acid (VPA) in Xenopus models for RP revealed varied outcomes depending on rhodopsin mutation. VPA improved degeneration associated with P23H rhodopsin, promoted rhodopsin clearance, and restored visual function. However, under light exposure, VPA exacerbated degeneration in the T17M mutant and showed adverse effects in T4K and Q344ter mutants, with outcomes similar to histone deacetylase (HDAC) inhibitors, suggesting effects of VPA was linked to autophagy regulation (55).

Rhodopsin is precisely trafficked to the OS of rod cells and transport defects cause autosomal dominant RP (46). A rhodopsin-photoactivated GFP-1D4 (RHO-paGFP-1D4) model was introduced in Xenopus to monitor rhodopsin trafficking in live cells, demonstrating that this model mirrors rhodopsin function and localization both *in vitro* and *in vivo*, enabling simpler and more comprehensive studies of rhodopsin and GPCR trafficking (56). *In vivo* studies in Xenopus identified that TBC1D32 is essential in retinal development and RPE differentiation, with deficiencies leading to disrupted cilia elongation, apical tight junction breakdown, loss of function, and an epithelial-mesenchymal transition-like phenotype, affecting photoreceptor differentiation and OS transport (57). FAM161A, a widely conserved microtubule-associated protein, has been linked to hereditary RP-associated blindness. Studies in Xenopus show that FAM161A has significant homology with Xenopus Tpx2, indicating a role in microtubule binding similar to Tpx2, laying a foundation for a molecular model of the FAM161A-microtubule complex (58).

##### 3.2.2.2 Genetic macular degeneration

North Carolina Macular Dystrophy (NCMD), a rare autosomal dominant disorder, arises from noncoding single nucleotide variants (SNVs) near *PRDM13* or duplications overlapping DNase I hypersensitive sites (near *PRDM13*/*IRX1*) (59–61). Researchers mapped interactions between *PRDM13* and *IRX1* promoters and identified 18 candidate cis-regulatory elements (cCREs), examining their activity via luciferase and Xenopus enhancer assays (59). Stargardt macular dystrophy, caused by mutations in *ELOVL4*, leads to macular degeneration and early-onset blindness. Overexpressing murine *ELOVL4* mutants in Xenopus rods showed that lack of the ER retention di-lysine motif led to mislocalization to Golgi and post-Golgi compartments, rather than the inner segment, altering photoreceptor structure and function and leading to retinal degeneration (62). Mutations in prominin-1 (prom1) have also been implicated in autosomal dominant Stargardt-like macular dystrophy, autosomal recessive retinitis pigmentosa, and cone-rod dystrophy (63). Prom1 expresses in the retinal outer segment (ROS) base, ROS patches, and cone outer segments (COS) in Xenopus and is also localized at the ciliary tips of multiciliated skin cells (64, 65). Mutations in the photoreceptor cadherin (PrCAD) lead to autosomal recessive cone-rod dystrophy (66). PrCAD is expressed in the retina and pineal photoreceptors and is localized to the ROS base in *X. laevis*, *Xenopus tropicalis*, and zebrafish retinas, with additional immunoreactivity observed at the ROS plasma membrane and the leading edge of COS disks (67). The mechanisms underlying outer segment (OS) malformations in prom1 and prCAD mutants remain unclear. Previous studies have shown that treating *X. laevis* eye cups with cytochalasin D induces OS malformations resembling those observed in prom1 mutants (68). Exploring the application of Xenopus model organisms may provide deeper insights into the processes of OS disk morphogenesis and the underlying mechanisms of prom1- and prCAD-related retinal degenerations (64).

##### 3.2.2.3 Color blindness and retinal tumors

Autosomal dominant congenital stationary night blindness (adCSNB) is caused by mutations in genes involved in the rod phototransduction cascade, including rhodopsin (RHO), transducin alpha subunit (GNAT1), and cGMP phosphodiesterase 6 beta subunit (PDE6B) (69, 70). In Xenopus, truncated PDE6B variants (PDE6β1–313, PDE6β1–314 fs50) localize to the phototransduction compartment, retain cGMP binding (non-catalytic), and constitutively activate the cascade by disrupting Pγ regulation, ultimately impairing rod adaptation (69). Complete color blindness links to CNGB3 mutations (e.g., F525N, T383fsX) that heighten photoreceptor susceptibility to cell death. Patch-clamp recordings in Xenopus oocytes expressing Wild Type or mutant channels revealed that these mutations enhance affinity for the activator CPT-cGMP, leading to cytotoxicity, particularly in the F525N variant, mitigated by CNG channel blockers or removal of extracellular calcium (71).

##### 3.2.2.4 Retinoblastoma and autosomal dominant vitreoretinal degeneration

Childhood eye tumors, such as retinoblastoma, are caused by biallelic inactivation of the *retinoblastoma 1 (RB1)* gene (72). By developing *rb1*/*rbl1* chimeric mutants using CRISPR/Cas9 in Xenopus embryos, researchers rapidly established a retinoblastoma model (73). Snowflake vitreoretinal degeneration, an autosomal dominant disorder, is linked to the *KCNJ13* gene encoding the *Kir7.1* inwardly rectifying potassium channel. The R162W mutation associated with this disease disrupts functional channel formation. Xenopus oocyte expression of mutant and wild-type Kir7.1 channels showed that the R162W mutation inhibits Kir7.1 activity by potentially altering phosphatidylinositol 4,5-bisphosphate (PIP2) gating (74).

### 3.3 Corneal development and disease model

#### 3.3.1 Corneal development in *Xenopus laevis*

The structural and developmental characteristics of the *X. laevis* cornea closely parallel those of humans (75). The development of the *X. laevis* cornea begins at stage 25 with the differentiation of a simple embryonic epidermis that overlies the developing optic vesicle. Around stage 30, after the detachment of the lens placode, cranial neural crest cells invade the space between the lens and embryonic epidermis, forming the corneal endothelium. At stage 41, a second wave of migrating cells containing presumptive corneal stromal cells infiltrates the stroma, leading to the formation of inner and outer corneal layers. These layers converge at a central stromal attraction center, which organizes radial fiber alignment. Post-stage 48, secondary stromal keratocytes migrate individually to the center, establishing the stromal layers. By stage 60, the stroma is extensively populated with collagen lamellae and keratocytes, and the stromal attraction center disappears. During early metamorphosis, the embryonic epithelium gradually transitions into adult corneal epithelium, characterized by microvilli coverage. By stage 62, the epithelium thickens, accompanied by extensive apoptosis in the epithelial cells, coinciding with eyelid opening. Post-metamorphosis, the adult frog cornea reaches its mature structure, comprising three cellular layers (epithelium, stroma, and endothelium) and two acellular layers (Bowman’s layer and Descemet’s membrane) (11, 76). Following initial maturation, the *X. laevis* cornea, particularly the stroma, continues to thicken and expand throughout the animal’s lifespan. In adult frogs, a *p63*-positive, wave-like structure at the limbus is observed, indicating corneal stem cell (CESC) activity. Proliferation assays reveal active division in basal corneal epithelial cells and limited proliferative activity in stromal and endothelial cells, suggesting ongoing corneal maintenance (11).

The larval corneal epithelium in *X. laevis* consists of three distinct layers: an outer epithelial layer, a basal epithelial layer, and a deeper fibrous layer containing major sensory nerve trunks. Basal epithelial cells express multiple pluripotency markers, including *sox2*, *p63*, *c-myc*, and *klf4*, indicating stem-like properties (77, 78). Notably, *p63* expression is confined to all basal epithelial cells, whereas c-myc is predominantly localized to a subset of basal epithelial cells and adjacent stromal tissue at the corneal periphery. Furthermore, *sox2* is expressed throughout the outer and basal epithelial cells, with higher intensity in a unique subpopulation of multinucleated or lobulated cells often located at the corneal periphery. Proliferation assays using thymidine analog EdU indicate that cell proliferation is concentrated in the basal epithelial layer and contributes to regeneration. Results reveal upregulation of pluripotency markers such as *sox2*, *p63*, and *oct60* during early lens regeneration, while immunostaining indicates a significant increase in *sox2*-expressing cells throughout the basal corneal epithelium within 4 h post-lens removal (78).

#### 3.3.2 Corneal disorders

##### 3.3.2.1 Hereditary endothelial dystrophies

*SLC4A11* is a member of the SLC4 bicarbonate transporter family expressed in corneal endothelial cells. Mutations in *SLC4A11* disrupt endothelial fluid transport, leading to dystrophies like congenital hereditary endothelial dystrophy (CHED) and Fuchs endothelial corneal dystrophy (FECD), but the precise pathogenic mechanisms are unresolved. *SLC4A11* encodes the bicarbonate transporter-related protein BTR1, and its mutations are associated with diseases such as CHED and Harboyan syndrome, characterized by vision and hearing loss. *SLC4A11*-mediated water transport is unique in its solute flux independence, inhibition by classic SLC4 inhibitors, and inactivity in CHED2 mutants (R125H). Studies in Xenopus oocytes and HEK293 cells demonstrate that *SLC4A11* facilitates water flux at rates approximately half that of aquaporins (79). Recent findings suggest that mSlc4a11 functions as an H(+)/OH(−) channel independent of co-transport with other ions, identifying OH(−) as its likely substrate. Moreover, mSlc4a11 activity is enhanced by extracellular and intracellular alkalinization (80–82). Using Xenopus as a model system, researchers have traced cellular lineages, examining the division patterns and fate of CESCs during development, homeostasis, and wound healing (83). Notably, a histologically confirmed Bowman’s layer is present in *Xenopus laevis*, which makes Xenopus a particularly relevant model for investigating a broader spectrum of human corneal pathologies, including those that primarily affect the epithelial-stromal interface (11).

##### 3.3.2.2 Peripheral corneal sclerocornea

The *RAD21 (R450C)* variant has been identified in a family with peripheral corneal sclerocornea. Microinjection of this *rad21* variant mRNA into Xenopus embryos disrupts the organization of stromal collagen fibers. Cells carrying the heterozygous *rad21* variant exhibit altered chromatin conformation and expression of cell adhesion genes, impairing neural crest migration and significantly reducing neural crest-derived periocular mesenchyme in the stromal region. These findings elucidate a mechanism by which the *RAD21 (R450C)* variant causes stromal defects (84).

##### 3.3.2.3 Limbal stem cell deficiency

LSCD, a debilitating condition caused by CESC damage or loss, has been modeled in Xenopus using transient psoralen-AMT treatment followed by UV exposure (PUV) (85). This model recapitulates LSCD features, including pigment invasion, corneal opacity, and neovascularization. PUV-induced depletion of *p63*-expressing basal epithelial cells promotes mitotic activity in residual cells, eventually restoring corneal transparency. This model provides a platform for understanding LSCD mechanisms and therapeutic strategies (86).

##### 3.3.2.4 Corneal stiffness disorders

Disordered stromal collagen fibers characterize corneal stiffening diseases (87). The *RAD21(C1348T)* variant is associated with such phenotypes, as shown by injection of wild-type and mutant *rad21* mRNA into Xenopus embryos. Mutant embryos exhibit disrupted stromal collagen fibers and reduced fiber diameter, which can be rescued by wild-type *rad21* overexpression. These findings highlight *RAD21*’s critical role in collagen organization and corneal development (88).

##### 3.3.2.5 Corneoscleral dysplasia

Corneoscleral dysplasia, marked by an indistinct corneoscleral boundary, involves *RAD21* mutations that disrupt neural crest cell migration through upregulation of *PCDHGC3* and *WNT9B* (89, 90). In Xenopus, *rad21* knockdown increases *pcdhgc3* and *wnt9b* expression, impairing neural crest differentiation. Suppressing *wnt9b* rescues migration defects, suggesting that *rad21* regulates corneoscleral boundary formation via WNT signaling modulation (91).

##### 3.3.2.6 X-linked megalocornea

Mutations in *Chordin-Like 1 (CHRDL1)* cause XMC, characterized by anterior segment enlargement, mosaic corneal dystrophy, premature cataracts, and glaucoma (92, 93). A novel *CHRDL1* frameshift mutation (*c.807_808delTC*) has been identified, leading to loss of function. Using Xenopus as a model, researchers demonstrated that *chrdl1* deficiency causes XMC-like phenotypes, linked to disrupted BMP signaling. Knockdown of *chrdl1* in Xenopus mimics human XMC, with reduced BMP receptor 1A and altered pSMAD1/5 phosphorylation. These findings implicate impaired BMP antagonism in XMC pathogenesis (94).

### 3.4 Lens development and disease model

#### 3.4.1 Lens development

In *X. laevis*, lens development can be categorized into five major stages, each defined by specific cellular and molecular events: In the early stages of development, the lens ectoderm thickens to form the lens placode, which begins to invaginate. By stage 27, the invaginated cell mass forms the lens vesicle primordium. At stage 32, the lens becomes polarized, forming a lens vesicle that separates from the sensory ectoderm. By stage 38, cells in the posterior portion of the lens vesicle elongate to form primary lens fibers, while the anterior cells develop into the epithelial layer. At stage 41, nuclear degradation begins, and primary fiber cells lose their nuclei. Between stages 44 and 48, secondary fiber cells differentiate in the equatorial region, forming concentric layers around the primary fibers, ultimately achieving a structure resembling the mature lens (76, 95).

#### 3.4.2 Congenital cataracts

Dozens of genes associated with congenital hereditary cataracts have been identified using animal models. These genes primarily encode lens proteins, connexins, membrane proteins, extracellular matrix components, cytoskeletal proteins, and transcription factors such as *FOXE3*, *HSF4*, *MAF*, and *PITX3* (96). Injection of low doses of morpholino oligonucleotides in *X. laevis* tailbud stages induces a subtle lens phenotype resembling cataracts (97). Knockout of mammalian homologs of cataract-associated genes, such as *TMEM114*, *CHRLD1*, *SIPAL3*, or *CELF1*, results in ocular phenotypes loosely associated with cataracts (98, 99). Inactivation of three genes in *X. laevis* via CRISPR/Cas9 revealed lens-specific defects. Mutants of the gap junction protein and nuclease *gja8* exhibited lens opacity, nuclear degradation, and fiber cell organization defects, while *dnase2b* mutants had normal external morphology but impaired fiber cell differentiation. The potential therapeutic effect of the chemical chaperone 4-phenylbutyrate was demonstrated by improving vision in *gja8* mutant tadpoles (96). *Connexin 50 (Cx50)* is one of the most commonly mutated genes associated with congenital cataracts. The *Cx50T39R* mutation exhibits dominant effects in *X. laevis*, resulting in significantly enhanced hemichannel currents, altered voltage gating, and induced cell death. All-atom molecular dynamics simulations revealed that the R39 substitution stabilized an open-state conformation of the N-terminal domain, leading to lens cytotoxicity and cataract formation (100). Mutations in RNA granule component *TDRD7* cause pediatric cataracts. In *X. laevis*, unilateral knockdown of *Hspb1* at stage 42 resulted in defects in eye and lens development, which were rescued by co-injection of mouse *Hspb1* mRNA (101). In a four-generation Chinese family with congenital cataracts, a novel missense mutation in the *MIP* gene, c.572C > G (p. P191R), was identified. Swelling assays in *X. laevis* oocytes showed that the P191R mutation reduced oocyte swelling rates by impairing MIP protein trafficking and decreasing its membrane localization, leading to cataract formation (102).

Channel Protein-Related Mutations: Membrane transporter monocarboxylate transporter 12 (*MCT12*), also known as creatine transporter 2 (*CRT2*), is expressed in the lens and associated with cataracts. Studies in *X. laevis* oocytes and human HEK293T cells revealed that multiple variants of the *SLC16A12* gene (p. Ser158Pro, p. Gly205Val, p. Pro395Gln, and p. Ser453Arg) significantly impaired creatine transport. Specifically, the p. Gly205Val and p. Ser453Arg variants failed to localize to the oocyte membrane, suggesting defective protein interactions during transporter processing (103). In autosomal dominant congenital cataract pedigrees, mutations in genes encoding various soluble and membrane proteins have been linked to lamellar cataracts. A novel mutation in the *MIP* gene (c.494G > A) caused substitution of a highly conserved glycine with aspartic acid (G165D) in aquaporin-0 (*AQP0*). Functional analysis of Xenopus oocytes expressing *AQP0-G165D* showed that the mutation disrupted *AQP0* trafficking and abolished water channel function. These findings highlight the essential role of *AQP0* in maintaining lens transparency and underscore how mutations in conserved residues of aquaporins impact their function (104).

### 3.5 Others

#### 3.5.1 Glaucoma

Using *X. laevis* oocytes as a modification and expression system, MβCD-induced currents were validated in oocytes expressing *TRPV4*. Further studies established the relationship between mechanical strain, free membrane cholesterol, the actin cytoskeleton, and stretch-activated transient receptor potential vanilloid isoform 4 (*TRPV4*) channels in human trabecular meshwork (TM) cells. These findings demonstrate that membrane cholesterol regulates the transduction of trabecular mechanical signals. This suggests that diet, cholesterol metabolism, and mechanical stress may influence conventional outflow pathways and intraocular pressure in glaucoma and diabetes, partially through TM mechanosensing (105).

#### 3.5.2 Galloway-Mowat syndrome

GAMOS is a rare disease characterized by early-onset nephrotic syndrome and microcephaly, often accompanied by various neurological features. Most cases are caused by pathogenic variants in genes encoding components of the KEOPS complex, including *OSGEP*, *TP53RK*, *TPRKB*, and *LAGE3*. Computational analyses revealed that three core members of the KEOPS complex—*Osgep*, *Tp53rk*, and *Tprkb*—are highly conserved across species, including *X. laevis*. RT-PCR and whole-mount *in situ* hybridization studies investigated the spatial and temporal expression patterns of *osgep*, *tp53rk*, and *tprkb* during early development in *X. laevis*. Results showed that these genes are expressed during early embryogenesis and are enriched in tissues and organs affected by GAMOS, including the developing eye (106). In a morpholino-mediated *tp53rk* knockdown model in *X. laevis*, depletion of endogenous *Tp53rk* led to abnormal eye and head development. This abnormal phenotype was rescued by expressing human wild-type *TP53RK*, but not by the c.163C > G mutant or another previously described GAMOS-associated mutant, c.125G > A (p. Gly42Asp) (107).

#### 3.5.3 Nuclear envelopathies

Mutations in nuclear envelope proteins are associated with various human diseases (nuclear envelopathies), particularly mutations in inner nuclear membrane proteins *emerin* and *MAN1*, which are linked to X-linked Emery-Dreifuss muscular dystrophy. Both proteins contain a highly conserved LEM domain known to interact with multiple transcription factors. Morpholino-mediated knockdown experiments in *X. laevis* showed that *MAN1* is critical for the development of tissues, including the eye. The effects of *MAN1* knockdown could be compensated by ectopic expression of *emerin*, restoring normal organogenesis. This highlights the essential role of *MAN1* in regulating genes critical for organ development and tissue homeostasis, supporting the necessity of LEM proteins in early embryonic development. Furthermore, *MAN1* deficiency may contribute to muscle- and retina-specific diseases (108).

#### 3.5.4 Ciliopathies

Regulatory Factor X (*RFX*) transcription factors are implicated in the pathogenesis of ciliopathies, severe human developmental disorders such as retinal degeneration. Studies on the *RFX7* gene in *X. laevis* revealed its expression in the eye, and knockdown of *RFX7* in Xenopus embryos led to defects in neural tube ciliogenesis and neural tube closure failure. *RFX7* regulates the expression of *RFX4*, a gene required for neural tube ciliogenesis. Ectopic expression of Foxj1, a key regulator of motile ciliogenesis, inhibited RFX4 expression but did not affect *RFX7* expression (109). Using *X. laevis* as a model, researchers rapidly evaluated the effects of novel compound heterozygous variants (p. Y503C and p. Q485*) in the centriole gene *PIBF1*, identified in Joubert Syndrome (JS) patients. The study indicated that JS results from a combination of amorphic and hypomorphic *PIBF1* alleles (110).

## 4 Discussion

The Xenopus system has emerged as an indispensable model for investigating ocular development and disease pathogenesis. Its unique embryonic transparency and experimental tractability have enabled precise characterization of *Pax6*-mediated eye field specification during early neurulation, revealing conserved genetic networks underlying vertebrate eye morphogenesis. Subsequent studies of corneal development have delineated the spatiotemporal dynamics of neural crest cell migration, providing mechanistic insights into stromal organization and endothelial differentiation. For disease modeling, CRISPR/Cas9 and TALEN approaches have successfully recapitulated human ocular pathologies including aniridia (*Pax6* mutations), retinitis pigmentosa (RHO variants), and congenital cataracts (GJA8 defects). Particularly noteworthy is the light-inducible RHO P23H model, which has provided unprecedented live visualization of photoreceptor degeneration dynamics through autophagy marker labeling.

While offering substantial advantages for developmental studies, the Xenopus system presents several constraints for clinical ophthalmology applications. The absence of macular anatomy precludes modeling of age-related macular degeneration, and the lack of intraocular pressure regulation mechanisms limits glaucoma research. Most disease models exhibit accelerated pathology compared to human chronic conditions - for instance, retinal degeneration typically progresses over days rather than years. Additional translational challenges include: (1) inability to perform standard visual function assessments (e.g., electroretinography, visual field testing); (2) limited capacity to study late-stage disease complications; and (3) absence of accessory ocular structures (eyelids, lacrimal apparatus) essential for investigating ocular surface disorders. (4) The species’ ancestral genome duplication event introduces functional redundancy that may obscure genotype–phenotype correlations, while its lack of macular specialization limits modeling of human macular pathologies.

Several strategic developments could enhance the model’s clinical utility. Establishing standardized visual behavior paradigms would facilitate functional assessment of therapeutic interventions. A tiered “Xenopus-to-mammal” validation platform could optimize drug discovery pipelines, leveraging Xenopus for rapid candidate screening followed by mammalian models for comprehensive evaluation. Particularly promising is the exploration of Xenopus’s remarkable regenerative capacity, which may reveal novel neuroprotective mechanisms applicable to retinal degenerative diseases.

## 5 Conclusion

In recent years, substantial progress has been made in studies related to eye development and associated diseases. Key breakthroughs include the identification of transcription factors and signaling pathway molecules that regulate the development of the lens, retina, and other ocular tissues. These findings have elucidated the genetic mechanisms underlying cataracts, glaucoma, and retinal degenerative diseases. However, the intricate gene regulatory networks governing eye development remain poorly understood, and the goal of organ-level eye regeneration remains a distant challenge. The *X. laevis* model, with its transparent embryogenesis, experimental flexibility, and efficiency in gene function studies, provides a unique platform for research in eye development. Integrating cutting-edge technologies with the Xenopus model offers promising opportunities to further uncover the molecular and genetic basis of eye development and congenital hereditary diseases.
